# Growing Communicators: A Fine‐Grained Analysis of Toddlers' Communicative Intentions From Requestive and Expressive, to Information Seeking and Giving

**DOI:** 10.1111/infa.70072

**Published:** 2026-02-25

**Authors:** Didar Karadağ, Gert Westermann, Marina Bazhydai

**Affiliations:** ^1^ Lancaster University Lancaster UK

**Keywords:** child‐caregiver interactions, communicative intentions, deictic gestures, information seeking, information transmission

## Abstract

Children readily respond to others' bids for communicative interactions from early childhood and actively initiate these themselves. However, the extent and variety of early child‐initiated communicative intentions is poorly understood, with theoretically derived intentions lacking systematic empirical support from naturalistic observations. This study, using a cross‐sectional data set, provides a fine‐grained characterization of communicative behaviors across three time points in the second year of life (13, 18, and 23 months, *N* = 47). We coded one‐hour‐long video recordings of home observations using a novel coding scheme to document the type of interactions toddlers initiated using four deictic gestures (reach, point, give, hold out) to meet a range of communicative goals, such as sharing interest, attention, or emotion, requesting an object or an action, seeking information or help, and giving information. Expressive interactions accounted for 49.9% of events, followed by requestive (40%), information/help seeking (8.3%), and information giving intentions (1.7%). These findings characterize early communicative toddler‐caregiver interactions and provide insights into the age‐related patterns of toddlers' propensity to seek and transmit information which emerge increasingly as part of toddlers' communicative repertoire.

## 1 Introduction

Children exhibit remarkable proficiency in navigating the intricacies of the social world right from the early stages of life. In infancy and toddlerhood, advancements in gesture production, language skills, increased mobility, and heightened socio‐cognitive awareness shape their social engagement. These developments lead children to engage with others in diverse ways and employ various communicative strategies (e.g., Karasik et al. 2011; Schneider and Iverson 2022), including actively initiating interactions to elicit specific responses from others. However, the extent and the rich variety of child‐initiated communicative interactions, and their underlying intentions, remains poorly understood despite rich theoretical debates (Southgate et al. 2007; Tomasello et al. 2007; Harris and Lane 2014; Harris et al. 2020). This is primarily due to research focusing on select subsets of behaviors or intentions studied in isolation (Oakes 2009). Here, we aimed to provide a more fine‐grained characterization of communicative behaviors and intentions in early childhood, with a particular focus on information‐seeking and even more so, information‐giving communications, both theorized to be present early in ontogeny and unique to humans, but not yet systematically documented in children's spontaneous behaviors.

### 1.1 Infants' Communicative Repertoire

Infants communicate with those around them using a mix of non‐verbal behaviors, like gestures and actions, and verbal behaviors, including non‐speech vocalizations and early language. Among non‐verbal behaviors, deictic gestures (i.e., pointing, holdouts, giving and reaching) play a crucial role in early communicative endeavors (e.g., Bates et al. 1975; Boundy et al. 2016, 2019; Cameron‐Faulkner et al. 2015). By around 4 months of age, infants actively use their hands to interact with objects, and by 6 months they reach for objects beyond their grasp (e.g., Bates et al. 1975; Rochat et al. 1999), especially when others are present, often alternating their gaze between the object and social partners (Caselli 1990; Ramenzoni and Liszkowski 2016). Nine‐ and 10‐month‐old infants start using holdouts and giving gestures with a potential intention to socially engage with others by sharing interest or attention with them (Boundy et al. 2016, 2019; Cameron‐Faulkner et al. 2015). These behaviors have been increasingly evaluated as precursors to index‐finger pointing—a hallmark of development associated with important linguistic and socio‐cognitive outcomes (Aureli et al. 2013; Blake et al. 1994; Carpenter et al. 1998).

Pointing, one of the most prominent deictic gestures used by infants from around 12 months (Tomasello et al. 2007), serves as a powerful tool for initiating interactions to elicit diverse responses from caregivers and other social partners. Infant‐initiated interactions triggered through pointing can be classified as aiming to request an object (e.g., Moore and D’Entremont 2001), to share or attract attention and interest (e.g., Liszkowski et al. 2004), to seek information or help (e.g., Begus and Southgate 2012; Kishimoto et al. 2007; for a review see Southgate et al. 2007), and to give information (e.g., Liszkowski et al. 2006, 2008). In each of these, the type of caregiver response elicited may differ qualitatively (e.g., handing the infant the desired object, providing the desired information/help). It can thus be argued that infants at 12 months have at least a rudimentary sensitivity with respect to the role of their own actions on their social partners' subsequent behavior to be able to direct the responses they would receive from others.

Despite the focus on pointing gestures in the study of early communicative development (e.g., J. Carpendale and Lewis 2006; Kuhn et al. 2014; LeBarton et al. 2015; Lucca 2020), earlier pre‐pointing gestures such as gives, hold‐outs and reaches also have been linked to developmental outcomes such as better expressive language. In their longitudinal study, Choi et al. (2021) found that at 10 months, show and give gestures better predicted 18‐month language skills than pointing, but by 14 months, pointing became the stronger predictor—suggesting that pointing gains predictive power as infants mature. Complementing this work, Ruether and Liszkowski (2024) demonstrated that caregiver pointing and infants' earlier showing behaviors predicted the age at which pointing emerged in infants aged 8–13 months. Overall, studies suggest a developmental continuum in infants' gestures and highlight the role of pre‐pointing gestures in the range of infants' communicative intentions (Boundy et al. 2019; J. I. Carpendale et al. 2021; Donnellan et al. 2020; Karadağ, Bazhydai, Koşkulu‐Sancar, and Sen 2024; Moreno‐Núñez et al. 2020; Perucchini et al. 2021; Salter and Carpenter 2022).

### 1.2 Infants' Communicative Intentions

Infants' intentions in communicative bids have been a topic of interest particularly in the context of pointing and almost exclusively with respect to two main communicative intentions: imperative (i.e., using an adult as a means to achieve a goal) and declarative (i.e., drawing attention to an interesting object or sharing interest with others; e.g., Bates et al. 1975; Camaioni 1997; Cameron‐Faulkner 2020). Crucial theoretical advancements have been made in extending research on infants' communicative intentions to account for arguably more complex interactions—information‐seeking and information‐giving (Begus and Southgate 2018; Harris and Lane 2014; Southgate et al. 2007; Tomasello et al. 2007).

With this more nuanced interpretation, infants' prelinguistic behaviors are viewed through the lens of shared intentionality and desire to exert reciprocal influences on each other's mental states in dyadic and triadic communications, and as a cultural learning and cultural knowledge transmission mechanism. If infants are capable of both intentionally requesting information from more knowledgeable others and sharing information with less knowledgeable others, by implication, this positions infants as active participants in the bi‐directional cultural knowledge exchange. This ability might be uniquely human given the lack of evidence that non‐human animals solicit information from and transmit knowledge to conspecifics (Bazhydai and Harris 2021; Burdett et al. 2017; Caro and Hauser 1992; Greenfield and Savage‐Rumbaugh 1993; Premack and Premack 1983; Rivas 2005; Strauss and Ziv 2012).

In this relatively recent line of work, infants have been shown to demonstrate non‐verbal information‐seeking behaviors even before they master language. By displaying inquisitive tendencies, infants start babbling (e.g., Goldstein et al. 2010), engage in actions such as showing or holding out objects towards others, giving and taking objects (e.g., Boundy et al. 2016), and social referencing employed to both seek social input (Striano et al. 2006) and gather information to resolve uncertainties (Bazhydai, Westermann, and Parise 2020; Goupil et al. 2016). Once available to them as a communicative tool, infants actively utilize pointing to inquire about objects that captured their attention (Begus and Southgate 2012; Begus et al. 2014; Lucca and Wilbourn 2018, 2019). The range of these prelinguistic behaviors, then, paves the way for the eventual development of sophisticated social information seeking using verbal questions (for reviews see Begus and Southgate 2018; Butler 2020; Harris et al. 2020; Lucca 2020).

Compared to imperative, declarative and information‐seeking intentions, infants' information‐giving intentions are substantially less studied. This might be due to information giving having traditionally been viewed as a subcategory of declarative intentions (Tomasello et al. 2007), or theorized to not emerge until the development of higher order cognitive capacities such as theory of mind (Bensalah and Caillies 2020; Caro and Hauser 1992; Davis‐Unger and Carlson 2008a, 2008b; Strauss et al. 2002; Ziv et al. 2016). Nevertheless, emerging research has documented that infants and toddlers do show information‐giving behaviors such as pointing at an object to inform an adult about its hidden location or demonstrating functions of an object to naive learners, although the scope of information sharing is limited to information pertaining to the child and the immediately accessible world around them in the here‐and‐now (e.g., Ashley and Tomasello 1998; Bazhydai, Silverstein, et al. 2020; Behne et al. 2014; Flynn and Whiten 2012; Karadağ, Bazhydai, and Westermann 2024; Karadağ, Bazhydai, Koşkulu‐Sancar, and Şen 2024; Karadağ et al. 2025; Knudsen and Liszkowski 2012a, 2012b; Liszkowski et al. 2006; Liszkowski et al. 2008; Meng and Hashiya 2014; O'Neill 1996; Vredenburgh et al. 2015). With development, the behavioral repertoire of information giving becomes more complex. By age three, children’s information giving becomes more structured and selective, evolving from simple demonstrations to more comprehensive teaching strategies that incorporate verbal explanations, rule reminders, and responsiveness to the learner’s needs (Strauss and Ziv 2012; Strauss et al. 2014). While information transmission is crucial for human cognition (Strauss and Ziv 2012) and cultural evolution (Caldwell et al. 2018), only a few studies have investigated the early emerging abilities in this domain, leaving a wide research gap spanning infancy and toddler years (for a review see Qiu et al. 2024).

### 1.3 Methodological Considerations

Early communicative intentions have been investigated in different ways. For instance, information‐giving and information‐seeking intentions have mostly been explored in lab‐based experimental studies (e.g., Bazhydai, Westermann, and Parise 2020; Begus and Southgate 2012; Liszkowski et al. 2006, 2008) compared to declarative and imperative intentions which have been also investigated in more naturalistic contexts (e.g., Cochet and Vauclair 2010b; Rowe and Leech 2019). Additionally, previous findings point to some inconsistencies between experimental longitudinal designs (Aureli et al. 2013, 2017; Camaioni et al. 2004; Carpenter et al. 1998; Perucchini et al. 2021) and more naturalistic studies (Cochet and Vauclair 2010b) where spontaneously generated behaviors are observed. For instance, in lab‐based studies, Camaioni et al. (2004) found that imperative pointing emerged at 11 months, preceding declarative pointing. In contrast, in other lab‐based work, Aureli et al. (2013, 2017) observed both intentions at 9 months and increasing similarly from 12 to 15 months; these results were also reflected in the findings of Perucchini et al. (2021). A longitudinal study conducted in experimental settings by Rohlfing et al. (2022) investigated infants' production and comprehension of imperative, declarative, and informative pointing between 12 and 18 months, comparing typically developing infants with those with language delays. While developmental trajectories differed slightly, by 18 months, both groups had reliably demonstrated both comprehension and production of pointing with each of the three types of communicative intentions. Compared to imperative and declarative, the informative pointing was first observed on average at 14 months, with all children successfully producing it when experimentally prompted by 18 months.

In more naturalistic studies, Cochet and Vauclair (2010a, 2010b) reported higher frequencies of declarative pointing in the second and third years, with an increase in declarative pointing with age. Thus, comprehensive studies of various communicative intentions are needed to reconcile the mixed findings and build upon them to provide a more nuanced picture of their developmental unfolding.

Recently, in parallel with the present study, two other studies embarked on this journey. Guevara et al. (2024) tracked 21 infants aged 7–13 months in naturalistic classroom settings where infants interacted with nursery caregivers and other children. This study found that early ostensive gestures, such as showing and giving, were related to the later emergence of pointing across diverse communicative contexts and partners. Using a microgenetic analysis of observations, six intentions were accounted for: imperative and declarative were most prevalent and observed most earliest observed (51% and 39%), phatic (to sustain communication) were observed substantially less frequently (5%), as well as and evaluative (to share joy or achievement, only observed in 0.2% of interactions). Interestingly, informative intentions through pointing were observed from 10 to 12 months and accounted for 2.7% of gestures, while information seeking was observed from 10 months and accounted for 1.4%.

Karadağ, Bazhydai, Koşkulu‐Sancar, and Sen (2024) investigated four communicative intentions through a wide range of verbal and non‐verbal behaviors initiated by infants and captured through natural home observations. These findings showed that at 18 months, Turkish infants actively initiated different interactions intended to achieve different communicative goals (e.g., requestive, expressive, information/help‐seeking, informative); infants most often initiated expressive interactions, followed by requestive and information/help‐seeking interactions. Finally, informative interactions were very rare in comparison to other interaction types and constituted less than 0.5% of all interactions initiated. This scarcity of information‐giving interactions is somewhat surprising given the results of the previous, lab‐based experimental studies which suggested that information transmission is an early emerging ability at as young as 12 months (e.g., Liszkowski et al. 2008), and the recent descriptive results from the classroom observation study (Guevara et al. 2024). Thus, we set out to investigate the early communicative interactions initiated by toddlers across the second year of life, with a special focus on characterizing the types of informative interactions.

In recent years, more research on infancy overall has shifted toward naturalistic settings, allowing observation and analysis of infant behavior in the context where most learning occurs initially—their homes (Oakes 2009, 2023; C. S. Tamis‐LeMonda 2023). This approach provides more ecologically valid data, accounting for individual differences influenced by factors like familial SES, communicative input from caregivers, and physical constraints of the infant environment (such as house size, available toys and objects at home, background sounds; e.g., Herzberg et al. 2022; Suarez‐Rivera et al. 2022, 2024; Swirbul et al. 2022). Unlike brief lab‐based studies, observing infant‐caregiver interactions over extended periods where these interactions tend to occur in short bursts (e.g., Herzberg et al. 2022; Slone et al. 2019) has the potential to capture nuances of early information giving that may be overlooked in carefully controlled lab settings (e.g., Bazhydai, Silverstein, et al. 2020; Karadağ, Bazhydai, and Westermann 2024; Liszkowski et al. 2008; Vredenburgh et al. 2015).

## 2 The Current Study

Based on the literature reviewed above, toddlers flexibly initiate different types of interactions to meet diverse communicative goals, such as sharing attention, requesting objects, seeking or giving information, but the existing empirical literature is lacking in studies documenting all four intentions in naturalistic contexts. Further, among these goals the one that received the least attention in prior research is information giving. The current study therefore addresses the broad distribution of communicative interactions at three time points in the second year of life and then focuses on the characteristics and prevalence of information‐giving interactions. We posed two research questions: (1) What is the prevalence and the age‐related patterns of four types of child‐initiated communicative interactions across 3 time points in the second year of life in natural settings, in particular information seeking and information giving? (2) What is the distribution of elicited and non‐elicited information giving among all informative interactions across the second year of life? Given the inconsistencies in the prior literature and a lack of studies of this type, this research was largely exploratory. Nevertheless, our predictions were as follows: Across the second year, children will spontaneously engage with their caregivers more to either share their interest or to request something from them compared to information seeking and information giving. We expected infants to both initiate information‐giving interactions and to respond by giving information to caregiver‐initiated information seeking; however, both types of information‐giving events were expected to be infrequent compared to other communicative intentions. Finally, we expected information giving to increase over the course of the 2nd year.

## 3 Method

### 3.1 Dataset

We used the “The Science of Everyday Play” (C. Tamis‐LeMonda and Adolph 2017) video dataset accessible to researchers registered on Databrary (https://nyu.databrary.org/volume/563). The primary dataset included home observation recordings of 63 toddlers in the second year of their lives. The nature of this dataset is cross‐sectional: toddlers from three different age groups (13‐, 18‐, and 23‐month‐olds) were recorded. Each toddler was recorded during two 2‐hour home visits.

For this study, we selected the first hour of the first visit for each age group (48/63 was publicly available at the time of coding) to account for external factors that might influence toddler behavior differently, such that the novelty due to the presence of others and being observed and followed at home would be consistent. The study design and data availability did not make it possible to carry out an a priori power analysis.

### 3.2 Participants

Data from 48 toddlers whose caregivers originally consented for the reuse of their first visit videos by another researcher was used in this study. There were 16 toddlers in the 13‐month age group (Mage = 13.00 months, SD = 0.18, Range = 12.7–13.2 months), 13 toddlers in the 18‐month age group (Mage = 18.00 months, SD = 0.19, Range = 17.6–18.2 months), and 18 toddlers in the 23‐month age group (Mage = 22.9 months, SD = 0.18, Range = 22.8–23.3 months). One toddler from the 13‐month group was later identified to follow an atypical trajectory that affected their gesture use; thus, we dropped the coded data from this participant, resulting in a final sample of 47 toddlers.

Data was collected from predominantly White, middle‐class families based in New York, USA. Most parents spoke to the infants exclusively in English (*n* = 29), while others also spoke another language (*n* = 18). Most parents reported that they received childcare support on a regular basis, either from a childcare center or someone else such as a nanny, babysitter, or relative (*n* = 32). Most mothers were White (*n* = 34) and reported having an education beyond a bachelor’s degree (*n* = 43). Most of the mothers were employed (*n* = 28).

The present study was conducted according to guidelines laid down in the Declaration of Helsinki, with written informed consent obtained from a parent or guardian for each child before any assessment or data collection by the original data owners. We received ethics approval (FST20112, dated May 11, 2021) from the Faculty of Science and Technology Research Ethics Committee at Lancaster University in the United Kingdom for the behavioral coding and re‐analysis of the primary dataset.

### 3.3 Primary Video Data Collection Procedure

When collecting the Science of Everyday Play video dataset, the following procedure was followed (C. Tamis‐LeMonda and Adolph 2017). The visits were made when the toddler and at least one of the caregivers were at home; some toddlers had other family members or house staff at home though these were not quantified. The toddlers and their caregivers were recorded using a portable video‐camera, and the observer mostly remained unresponsive to toddler's communicative bids toward them to avoid interfering with the natural behavior of the dyad. Additionally, during these visits, the caregivers were instructed to continue their daily lives as they normally would and not to interact with the observers unless there were situations where they could not avoid the interaction (such as when they requested recording to be stopped for privacy reasons during feeding or changing their children). However, if the toddlers referenced the observers such as by pointing towards them or asking them to do something with them, the parents acknowledged these requests and explained why the observer cannot engage (e.g., she will eat later, she is recording, etc.).

During home observations, toddler‐caregiver dyads engaged in various activities in their everyday environment. These activities included mealtime (where children were fed, or they had snacks and drinks), screen‐based activities (where they watched TV together with their parents or looked at photographs or videos from their parents' phones or tablets), and playtime (where the dyad played with toys, listened to music, and danced together, played physical games such as *catch the ball*, etc.). Additionally, the home observations encompassed household activities where the caregivers carried out their daily chores in the presence of their children or by involving them in these tasks such as when they were cooking, folding the laundry, tidying up the toys, unloading the dishwasher, or making coffee. Finally, among the most common play activities were reading books with the caregivers, drawing and coloring, playing with blocks, making puzzles, listening to music, and dancing.

### 3.4 Behavioral Coding

We adapted a coding scheme developed by Karadağ, Bazhydai, Koşkulu‐Sancar, and Sen (2024), coding toddler behaviors in an event‐based manner (e.g., Bornstein et al. 2020). The camera always followed the toddler, and we coded toddlers' interactions when they were in the presence of or in communication with another person. An event was defined as an interaction that started with a trigger behavior from the toddler, such as pointing to an object, and ended when the query associated with the trigger behavior was resolved or when either the parent or the toddler disengaged from communication. All behaviors initiated within 3 s following the toddler‐initiated trigger behavior were coded. This time frame was chosen based on the previous literature on contingency in mother‐infant interactions (e.g., Kuchirko et al. 2018; C. S. Tamis‐LeMonda et al. 1998, 2013). In this study, unlike in the previous coding protocol that looked at a broader range of behaviors (Karadağ, Bazhydai, Koşkulu‐Sancar, and Sen 2024), we only focused on toddlers' use of deictic gestures as the most prevalent non‐verbal behaviors to initiate an interaction.

A trigger behavior (i.e., toddlers' pointing, giving, reaching, holding out gestures) was coded as marking either toddler‐ or caregiver‐initiated interaction. In the following cases, the interaction was coded as toddler‐initiated: (1) if the toddler behavior did not occur within 3 seconds of the last caregiver‐initiated behavior (e.g., the mother asks the toddler to look at something, the toddler points 10 s later); (2) if the toddler disengaged from the previous interaction with a new trigger behavior (e.g., the mother asks the toddler to look at the window and the toddler gives an object to the mother). If the toddler behavior occurred within the 3 seconds of the last adult‐initiated behavior (e.g., the mother asks the toddler to give her an object, and the toddler responds within 3 seconds), the event was instead coded as caregiver‐initiated. In rare cases where the 3 seconds prior to the trigger behavior were not informative in terms of identifying the initiator of the interaction (e.g., toddler pointed to an object, but it was in response to the parent's question 4 seconds ago), we extended the time frame to 5 seconds (e.g., when the child walked towards and gave an object to their mother; due to the walking pace of the child 3 s before the giving action was not informative to define who initiated this behavior whether the mother requested the object or the child wanted to give the object to the mother).

The coding was completed in two steps: The first step included identification of the events and event‐related characteristics; the second step included the coding of the perceived communicative intent of the events.

#### 3.4.1 First Step Coding

**FIGURE 1:**
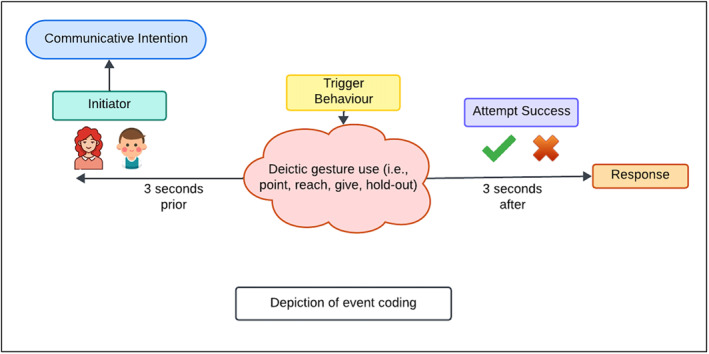
Depiction of event coding.

The aim of this step was to identify trigger events that fit the criteria as specified above. Once we identified an event, we coded three tiers for each event. The first tier was called “Initiator”, and it represented whether the interaction was initiated by the caregiver or toddler. The second tier was called “Bid Success” and it represented if the bid had been responded to appropriately or not (e.g., successful bid: the toddler pointed to some object to request it, the mother looked at it but responded as “No, you can't get it”/“Here, it is.”; failed bid: the toddler pointed to some object to request it, the mother did not notice nor acknowledge the point, instead showed something else and said “Look at this!”). It is important to emphasize that a bid was evaluated as successful even if it did not achieve what it set out to achieve (e.g., receiving an object after pointing to that object to get it). We reasoned that if the communication between the communicative partners continued to flow and the interlocuters acknowledged each other, whether the initial aim of the bid was achieved or not was irrelevant (Clark 1996; Grice 1975). The third tier was called “Trigger Behavior”, and it represented the type of behavior that was used by the toddler to initiate the interaction or to respond to the caregiver's bid (Figure 1).

Trigger behaviors were chosen based on the previous literature on early non‐verbal communicative interactions initiated by toddlers. We coded all instances where toddlers produced pointing (to an object, location, or a person), hold‐out, giving and reaching gestures in the presence of or in close proximity to a social partner. We defined these behaviors as follows:

3.4.1.1

- *Pointing*: Using either index‐finger or full hand toward a particular object or a social other. In addition, we included behaviors in this category that were not precisely “pointing” but functioned as pointing (such as a toddler sitting on a couch looking at their parent and tapping on the couch with both hands to request their mothers to sit with them). We did not code instances in which toddlers' index fingers appeared to be pointing but were instead used to press on an object, touch a surface, or scratch at it.
- *Hold‐out*: Picking an object in one or both hands and holding it out toward the social partner.
- *Giving*: Holding an object in one or both hands and dropping/handing over the object in the social partner’s hand/lap, placing the object near the other, or placing an object to a location requested by the partner (such as when tidying toys, putting toys in a basket that the parent is holding towards the child).
- *Reaching*: Extending one or both arms toward an object or a social partner including cases where children reached up to the parent to be picked up, to take an object from the parent and to aid their own mobility.

3.4.1.2

The instances of these deictic gestures were not coded in the following cases: (1) when the toddler was alone, (2) when the toddler behavior was directed to the observer (e.g., they looked at and gestured toward the observer, and did not acknowledge the parent), (3) when the toddler and the caregiver were in the same room but the caregiver was not in view of the camera recording and the interaction between the toddler and the caregiver could not be reliably predicted (e.g., the toddler gestured toward the caregiver, but the coder could not see or hear the caregiver), (4) when the toddler and the caregiver were together (sitting on a play mat in close proximity) but the toddler behavior was not directed to the caregiver (they are examining objects or reaching to objects without acknowledging the parent).

#### 3.4.2 Second Step Coding

**FIGURE 2:**
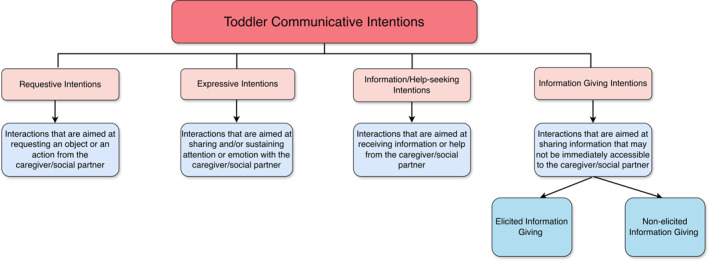
Types of interactions classified by the perceived intention of the interaction for the second step coding procedure.

Once all events and event‐related characteristics were reliably identified in the video dataset, all videos were coded again to identify the perceived intention of the interactions (Figure 2). This was coded in one tier which was called “Interaction Type” and could be *requestive*, that is, requesting an object or an action from the social partner, *expressive*, that is, aiming to either share emotion about an object or an action, or attract, share or sustain attention of a social partner, *information/help seeking*, that is, aiming to seek information or help from the social partner, and *information giving*, that is, aiming to transmit information to the social partner. As infants’ communicative intentions are often difficult to assess objectively in naturalistic settings, we followed a conservative approach. In instances where the contextual information was not sufficiently clear to support a different intention, the events were coded as expressive by default. Expressive intentions were identified through affiliative cues such as mutual gaze, affect, or play‐like actions. Conversely, events were classified as requestive or information‐seeking only when substantiated by additional evidence, including persistence, repetition, or the child’s response to the caregiver.

In order to characterize and understand the development of information giving across the second year of life, information giving events were classified into two categories as non‐elicited/spontaneous information transmission and elicited information transmission. The first category included events where toddlers shared information that was opaque to their caregivers spontaneously, such as telling them about something that happened in their absence, exchanging looks with their caregiver and pointing to something that only they can see or information about their own mental and physical states (e.g., pointing to their finger and saying “hurt”). For the second category, we coded toddlers' information‐giving responses to their parents' genuine information‐seeking questions where the response to the posed question was not immediately clear to the parent. For instance, if a parent asked the toddler “Where is X?” and if the parent did not have visual access to the X at that moment, or if the parent asked the toddler “Which one do you want? This one or this one?” by holding two objects towards the toddler, the toddlers' responses to these questions were coded as information giving as the toddler had an opportunity to provide specific information to the parent. However, not all parents' questions aimed to seek information. For example, if the parent asked, “Could you please hold this?”, the toddler's response to this question was not coded as information giving because the question was not a genuine information‐seeking attempt but rather a polite request from the caregiver. Similarly, if the parent and toddler shared the same visual field and the parent asked a question about aspects of the context that were accessible to both (e.g., the parent and toddler are looking at a picture book and the parents asks, “Where is the ball?”, “How many are there?”), the toddlers' responses were not coded as information giving because of the pedagogical nature of such questions where the parent aimed to guide the toddler's learning rather than learn from the toddler (e.g., Yu et al. 2019).

Finally, the intentions of the interactions were assigned based on the initiator of that event. If the toddler showed a give gesture because the parents asked for an object from the toddler by holding their hands toward them, this interaction was coded as *requestive* because the gesture of the toddler was initiated by the caregiver. Besides describing an overall distribution, we did not analyze the parent‐initiated interactions in this study.

### 3.5 Training Coders and Interrater Reliability

The videos were coded using ELAN (ELAN 2023; Wittenburg et al. 2006). Following the coding procedure, we conducted a reliability analysis in two steps with two different secondary blind coders. The first author acted as the secondary coder in the first step and as the main coder in the second step. First, the coders identified the events based on the developed event definitions while watching the first hour of a free‐flowing video. Following the identification of the event, coders moved onto coding other event related characteristics such as initiators, attempt success and trigger behaviors. In the second step, a new blind coder was trained to code the type of interactions for the reliability analyses.

We randomly selected 25% of the data to be coded for reliability. In the first step, after the coders coded the videos independently, they discussed each case that was coded by only one of them to make sure the coded interactions followed the criteria of this study. In this agreed‐upon list of events, 77% of all events were reliably identified by both coders.^1^ Categorical variables showed substantial to excellent agreement (all kappas ranged between 0.67 and 0.89 [*κ*_initiator_ = 0.67, *κ*_bid success_ = 0.77, *κ*_trigger behavior_ = 0.89], % agreement rate ranged between 84% and 93%) and Intraclass correlations calculated for the continuous variable (*r* = 0.77) showed good reliability. In the second step, coders showed substantial agreement (kappa = 0.75, % agreement = 85%). After sufficient reliability was reached, the remaining data were coded independently by the primary coder. A potential bias that could emerge from the first author coding a significant portion of the data was mitigated through the implementation of a two‐step coding process, accompanied by two independent reliability coders. The outcomes of this process demonstrated consistency with prior research (for reviews, see Tomasello et al. 2007; Guevara and Rodríguez 2023).

## 4 Results

### 4.1 Event Characteristics

Overall, we identified a total of 4440 events (*M* = 54.44, SD = 38.2, Range: 30–208). Approximately 64% of all events coded were initiated by the toddlers with one of the trigger behaviors, in the remaining 36% of the events, toddlers' use of the trigger behaviors was as a response to parents' bids (caregiver‐initiated). Since the focus of the current paper is toddler‐initiated behaviors, our reporting below is based on toddler‐initiated events (except for “elicited information giving” which occurred as response to parent's information‐seeking questions/attempts and allows us to characterize this communicative intention more fully as manifested in infants' everyday environments).

In total, we identified 2817 events initiated by the toddlers (*M* = 55.41 per 1‐hour, SD = 38.99, Range: 17–175). An event lasted 3.23 s on average. Among these, 98% were coded as successful (*n* = 2754). Toddlers used all four trigger behaviors to varying degrees with reaches being the most frequent, followed by points, gives, and holdouts (see Table 1).

**TABLE 1:** Distribution of trigger behaviors in toddler‐initiated interactions.

Trigger behavior	Number of events	% of total events
Reach	913	32.4
Point	856	30.4
Give	619	22.0
Hold‐out	429	15.2

Across all age groups, all four types of events based on perceived communicative intent were initiated at least once. *Expressive* events were initiated most frequently, followed by *Requestive* and *Information/Help Seeking* interactions. Even though rare compared to other intentions, toddlers also initiated *Information Giving (Non‐elicited)* events (See Table 2).

**TABLE 2:** Distribution of communicative intentions in toddler‐initiated interactions.

Interaction type	Number of events	% of total events
Expressive	1407	49.9
Requestive	1128	40.0
Information/help seeking	235	8.3
Information giving	47	1.7

**FIGURE 3:**
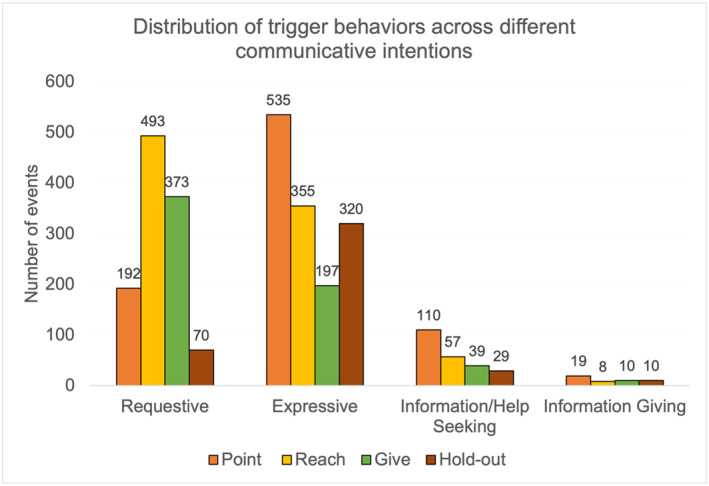
Distribution of trigger behaviors across different communicative intentions.

As evident in Figure 3, pointing was most frequently used to initiate expressive events, and the most prevalent among information/help seeking events, whereas requestive events were most frequently initiated with a reach and a give.

### 4.2 Age‐Related Patterns of Toddler‐Initiated Communicative Intentions

We present the proportion of each type of interactions initiated by toddlers across age groups in Table 3. Since the assumption of normality was not met, by using the proportions, we conducted four Kruskal–Wallis tests to investigate the role of age across each communicative intention while applying Bonferroni correction for multiple tests. The results of these tests do not show significant differences across age groups, all *p*s were bigger than the Bonferroni adjusted critical alpha value of 0.0125 (requestive *p* = 0.022, expressive *p* = 0.202, information/help seeking *p* = 0.875, information giving *p* = 0.260). We further explored the relationship between age as a continuous variable and each type of interactions by calculating correlations. The results showed that only information giving showed a significant positive correlation with age *r*(45) = 0.284, *p* = 0.026 (one‐tailed). Since it was not possible for us to conduct an a priori power analysis and the post hoc power analyses are of limited value (Heinsberg and Weeks 2022; Lakens 2022; Zhang et al. 2019), we performed a sensitivity analysis to determine Minimum Detectable Effect Size (MDES). This test revealed that the fixed study parameters (*N* = 47, 4 groups, *α*_Bonferroni‐adjusted_ = 0.0125), were only sufficiently powered (at 80%) to detect a large effect size (Cohen's *f* = 0.60). While we can confidently rule out large age‐related effects, these specific results should be interpreted with caution regarding smaller effect sizes, which would require larger samples to detect reliably.

**TABLE 3:** Proportions of communicative interactions initiated by each age group.

	Age group	Mean	SD	*N*
Requestive	13‐month	0.47	0.16	16
	18‐month	0.43	0.12	13
	23‐month	0.35	0.09	18
	Total	0.42	0.14	47
Expressive	13‐month	0.47	0.15	16
	18‐month	0.50	0.14	13
	23‐month	0.54	0.09	18
	Total	0.50	0.13	47
Information/help seeking	13‐month	0.05	0.03	16
	18‐month	0.06	0.08	13
	23‐month	0.08	0.10	18
	Total	0.07	0.08	47
Information giving	13‐month	0.006	0.01	16
	18‐month	0.009	0.01	13
	23‐month	0.02	0.03	18
	Total	0.01	0.2	47

**FIGURE 4:**
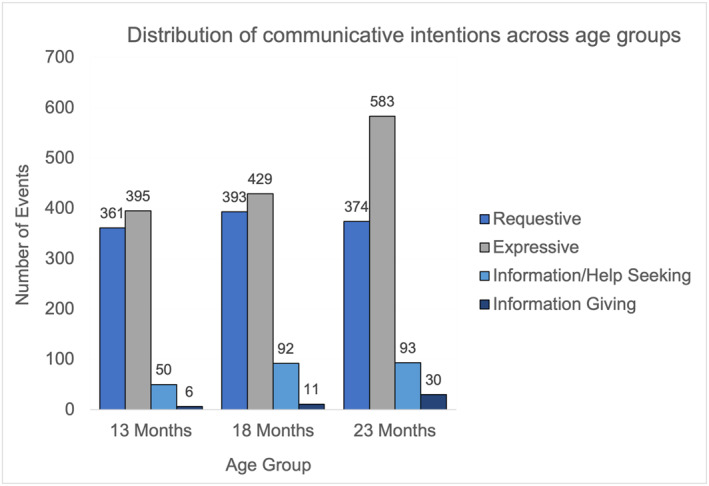
Total number of events initiated for each communicative intention across age groups.

As can be seen in Figure 4, the distribution of communicative intentions remained similar across the three age groups: toddlers initiate more interactions with expressive intention followed by requestive, information/help seeking and finally information giving, however, as toddlers get older, the number of events initiated with each communicative intention shows an increasing trend.

### 4.3 Toddlers' Information Giving

To collectively account for all events that we characterized as information giving in our coding scheme, we created a new variable—combined information giving where we summed up the number of elicited information giving events and the number of non‐elicited information giving events. Based on this combined information giving measure, 40 out of 47 toddlers in the sample displayed at least one informative event during an hour‐long interaction with their caregivers. Eighteen out of 47 toddlers showed at least one toddler‐initiated (non‐elicited) information giving event and 38 out of 47 toddlers showed at least one elicited information giving event.

### 4.4 Age‐Related Patterns of Elicited and Non‐Elicited Information Giving Behavior

Previous non‐parametric tests showed that toddler‐initiated (non‐elicited) information giving was present from 13 to 23 months consistently, when treating age as a categorical variable. Here, to explore the relationship between age and information giving more specifically, we calculated correlations between age as a continuous variable and each type of information giving as well as combined information‐giving scores (for descriptive statistics, see Table 4). We found that age as a continuous variable was positively correlated with non‐elicited information‐giving, *r*(45) = 0.284, CI [0.01, 0.52], *p* = 0.026 (one‐tailed), with elicited information‐giving, *r*(45) = 0.252, CI [−0.02, 0.49], *p* = 0.044 (one‐tailed), and with combined information‐giving score, *r*(45) = 0.306, CI [0.03, 0.54], *p* = 0.018 (one‐tailed). Taken together, the results indicate some evidence for the existence of different types of information giving across the second year of life, potentially increasing with age. These correlations represent small‐to‐medium effect sizes, providing evidence that different types of information giving emerge and potentially increase across the second year of life. However, given the relatively modest sample size (*N* = 47), these effect size estimates should be interpreted cautiously.

**TABLE 4:** Mean number of different information giving events different age groups.

	Age group	Mean	SD	Min	Max
Non‐elicited information giving	13	0.38	0.81	0	3
	18	0.85	1.07	0	3
	23	1.667	2.83	0	11
	Total	1.00	1.94	0	11
Elicited information giving	13	2.31	2.57	0	8
	18	5.46	3.64	1	13
	23	4.67	4.26	0	13
	Total	4.09	3.75	0	13
Combined information giving	13	2.69	2.92	0	8
	18	6.31	3.86	1	13
	23	6.33	6.21	0	24
	Total	5.09	4.89	0	24

## 5 Discussion

In this study, we had two overarching aims: First, we investigated theoretically derived toddler‐initiated communicative intentions across three time points cross‐sectionally during the second year of life. Second, we focused on the information‐giving intention to characterize the distribution of both elicited (i.e., initiated by the caregivers) and non‐elicited information transmission events. We found that toddlers, as predicted, actively initiated communicative interactions with a range of communicative intentions (requestive, expressive, information/help seeking, information giving) using four deictic gestures. Age was not related to the propensity to generate each type of the intentional interactions. Almost all of the communicative bids from toddlers received an appropriate response from the parent. Additionally, although it was much less frequent in occurrence, we found that 1/3 of the toddlers initiated at least one non‐elicited information‐giving event. When this was combined with the elicited information giving which occurred as a response to parents' information seeking requests, 85% of all children displayed information giving. Here, there was a positive relation between age and the number of information‐giving events: as toddlers got older, their information giving increased.

The findings of the current study build upon and extend the findings from previous literature (e.g., Guevara et al. 2024; Karadağ, Bazhydai, Koşkulu‐Sancar, and Sun 2024, Rohlfing et al. 2022; Salo et al. 2019; see Guevara and Rodríguez 2023 for review) in several ways. By incorporating the less often studied deictic gestures such as hold‐outs, gives and reaches in addition to the more commonly investigated pointing gesture, and by using a wide range of theoretically‐derived communicative interactions, we provide a fine‐grained characterization of communicative behaviors across the second year of life using natural home observations cross‐sectionally at three time points. Additionally, we show that while still being much less frequent compared to other communicative interactions, information giving emerges increasingly as part of toddlers' communicative repertoire. Such detailed and comprehensive exploratory investigation helps paint a richer picture of communicative experience and development embedded in children's natural environments (Oakes 2009, 2023; C. S. Tamis‐LeMonda 2023). Toddlers emerge as actively generating communicative interactions to not only use others to request objects and share attention, but also solicit and propagate information to others in the context of active social learning. Overall, this work provides empirical support for the early emergence of the information‐seeking and information‐giving intentions, positing children's active role in the process of cultural knowledge exchange from infancy (Bazhydai and Harris 2021; Begus and Southgate 2018; Burdett et al. 2017; Harris and Lane 2014; Harris et al. 2020; Southgate et al. 2007; Strauss et al. 2002; Strauss and Ziv 2012; Tomasello et al. 2007).

Here, toddlers initiated 55 events on average during an hour‐long observation in their home settings, using different deictic gestures. In terms of communicative intentions, toddlers most frequently initiated *expressive* intentions—to share or sustain attention, intention, or emotion with caregivers. The second most frequent communicative intention was *requestive*—to request an object or an action from caregivers. These two intentions accounted for almost 90% of all interaction initiated by the toddlers in this sample. The prevalence of these intentions was similar to what has previously been observed in a similar paradigms in two different cultures (Guevara et al. 2024; Karadağ, Bazhydai, Koşkulu‐Sancar, and Sen 2024). Additionally, these findings are also in line with the prior literature which mainly focused on imperative and declarative intentions (akin to what we characterized as “requestive” and “expressive”; for reviews, see Tomasello et al. 2007; Guevara and Rodríguez 2023).

While it was substantially less frequently observed (just over 8% of all interactions, approximately 5 events per hour), toddlers also initiated interactions with the intention of receiving information or help from their caregivers. This finding crucially corroborates a limited set of prior studies showing that children initiate information and help seeking as part of the emerging repertoire of explorative behaviors (e.g., Harris and Lane 2014; Ronfard et al. 2018). While these behaviors have attracted researchers' attention in the past 2 decades, the majority of a limited set of studies that focused on early information seeking were conducted in controlled laboratory settings (e.g., Bazhydai, Westermann and Parise 2020; Begus and Southgate 2012; Begus et al. 2014; Goupil et al. 2016; Lucca and Wilbourn 2018) in comparison to an even smaller number of studies conducted in natural or naturalistic settings (Boundy et al. 2019; Chouinard et al. 2007; Guevara et al. 2024; Karadağ, Bazhydai, Koşkulu‐Sancar, and Sen 2024; Olson and Masur 2013). For instance, Chouinard et al. (2007) conducted a diary study aimed at exploring how preverbal children, who lack the ability to verbally formulate questions, seek information from their caregivers. To accomplish this, the researcher trained parents to distinguish different types of nonverbal behaviors such as gestures and non‐speech vocalizations in order to identify instances where their children were attempting to communicate inquiries. Parents, then, tracked children's non‐verbal attempts at seeking information from their caregivers. Chouinard et al. (2007) found that 12–18‐month‐old toddlers sought information from adults through the use of gestures as well as information‐seeking vocalizations. In an experimental study, 16‐month‐old toddlers were more inclined to seek information about novel objects through pointing from an adult who had previously consistently offered reliable information in comparison to an adult who had been inconsistent or unreliable (Begus and Southgate 2012). Recently, Karadağ, Bazhydai, Koşkulu‐Sancar, and Sen (2024) found that 18‐month‐old Turkish infants used a wide range of verbal and nonverbal behaviors (e.g., vocalizations, deictic gestures, action demonstrations, non‐specific play actions) to initiate interactions with their social partners with the intention to receive information from them. Similarly, Guevara et al. (2024) reported that from 10 months, infants displayed information seeking though ostensive and pointing gestures, though at a very small rate until 12–13 months of age. Our findings further elaborate that spontaneous information/help seeking is present as early as 13 months and observed reliably across the second year of life, and therefore not only complement but extend the previously reported findings on early manifestations of information‐seeking behavior.

The second focus of our study was on early information giving that we reasoned would reliably manifest itself in the second year of life. Previous studies conducted in laboratory settings demonstrated that while infants and toddlers share information following explicit requests or cues regarding the recipients' episodic knowledge states based on visual access through pointing and action demonstrations (Bazhydai, Silverstein, et al. 2020; Behne et al. 2014; Liszkowski et al. 2006, 2008; Karadağ, Bazhydai, and Westermann 2024; Karadağ et al. 2025; Rohlfing et al. 2022; Vredenburgh et al. 2015), they are less likely to spontaneously initiate such interactions without being prompted (Guevara et al. 2024; Karadağ, Bazhydai, Koşkulu‐Sancar, and Sen 2024). One reason for this might be that in lab settings, the tasks are specifically designed to create a situation where the child is more likely to be “in the know,” whereas during the natural flow of daily events in their homes, these sorts of situations are less likely to happen. This is supported by the study conducted in the experimental lab setting by Rohlfing et al. (2022), reporting that 40%–50% of 12‐month‐olds demonstrated comprehension and production of informative gestures, while 100% did so at 18 months. In line with our findings, however, infants' imperative and expressive gestures were demonstrated by about 80% at 12 months, following a similar pattern of prevalence as we found in the present study: imperative emerging first, expressive second and informative last (note that this study did not include information‐seeking gestures).

In light of these core methodological differences, the relative infrequency of information giving is not surprising. While interpreting intentions behind communicative interactions initiated by toddlers is challenging on its own, it is especially difficult when it comes to providing information to others. In prior work, toddlers' information provision was typically prompted by adults looking for objects through reaching, looking, self‐directed questions (e.g., Liszkowski et al. 2006, 2008), or explicitly requesting information from toddlers (e.g., Bazhydai, Silverstein, et al. 2020; Vredenburgh et al. 2015). However, in the absence of these cues or when the adults do not explicitly signal these to toddlers, categorizing these behaviors as information giving becomes challenging. This difficulty arises because it relies on making rich assumptions about toddlers having metacognitive awareness (e.g., realizing they possess unique information) and theory of mind skills (e.g., understanding others' needs) when in reality they may not have yet developed these capacities, and may not need these at all in order to effectively transmit information to others (Bazhydai and Harris 2021; Bazhydai and Karadağ 2022; Kulke et al. 2018).

By taking this caveat into consideration, our methodological approach was to also code information giving where toddlers provided information in response to their caregiver's genuine information‐seeking questions. These were situations where the responses to questions were not immediately accessible to the caregivers such as when they lacked visual access or when the response pertained to internal states of the toddler (e.g., their preferences). Notably, Guevara et al. (2024) similarly coded infants' informative gestures as those occurring in response to caregivers' open‐ended questions, such as “Where are the books?”. Informative points accounted for a total of 2.7% communicative interactions in their dataset. In our study, we found that toddlers overall displayed more elicited compared to non‐elicited information behavior and that there was a positive relation between age and different types of information giving; however, given the relatively small sample size in each age group, it is difficult to make strong conclusions.

Previous relatively scarce research on early information giving has primarily centered on pointing behavior (e.g., Behne et al. 2014; Guevara et al. 2024; Knudsen and Liszkowski 2012a, 2012b; Liszkowski et al. 2006, 2008; Meng and Hashiya 2014) and has been grounded in two key assumptions. The first assumption posits that even infants possess the ability to efficiently monitor both their own and others’ epistemic states, enabling them to discern differences in knowledge levels. Some evidence supports this, with studies suggesting that infants can track their own (Bazhydai, Westermann, and Parise 2020; Goupil et al. 2016) and others’ (Tomasello and Haberl 2003; Surian et al. 2007) episodic knowledge states. However, the extent to which these abilities motivate infants’ communicative intentions, particularly in the context of information giving, remains insufficiently established. In our study, while we presume that toddlers initiate these interactions with an intention to give information, we do not claim that toddlers consciously reason that they possess unique information that needs to be transferred. Instead, the intention to transmit information (akin to proto‐teaching, Strauss and Ziv 2012) might precede the intention to teach observed in older children, and do not necessarily need to be motivated from components of theory of mind that may arguably not yet be present in toddlers (e.g., Kulke et al. 2018; Poulin‐Dubois et al. 2018).

Regarding the second assumption, which posits that children exhibit a prosocial motive to provide information when others want or need it, recent findings from instrumental helping literature challenge this notion (Dahl and Paulus 2019; Paulus 2020). While infants may indeed have a prosocial inclination to help others, recent studies suggest that, in the case of 18‐month‐old children, their helpful behavior is not solely driven by the specific needs of the recipient. Instead, their motivation seems to stem from a broader desire to engage in social interactions and be part of social dynamics (Paulus 2020) as well as from a motivation to complete goal‐directed actions that are not completed by others (Michael et al. 2022). Considering these findings, we suggest that incorporating observational studies that take place in toddlers' home environment as they continue their daily routine over extended periods are particularly useful. By doing so, we can build alternative pathways to understanding the ontogeny of early information transmission without relying on these higher‐level assumptions.

While the current study provides valuable insights into early communicative interactions, specifically with an inclusion of information seeking and transmission, these results should be treated as hypotheses‐generating due to several methodological limitations. As this was secondary data analysis, we had no control over how original observations were conducted, the sample size or the sample characteristics, which might have impacted our systematic coding and the resulting conclusions. As the data was cross‐sectional, it did not allow for investigating individual differences in developmental change. For instance, we could not account for parental behaviors, characteristics or attitudes, such as scaffolding or instead hindering child‐initiated interactions. Crucially, while the results of our analysis of the present cross‐sectional dataset suggest an age‐related interindividual pattern consistent with a possible developmental trajectory, future research would greatly benefit from using longitudinal datasets including age groups up to 4 years to explore the intraindividual changes in these interactions, and the role, or lack thereof, of higher‐level cognitive capacities such as ToM or metacognition. Thus, longitudinal conclusions should not be drawn from this cross‐sectional data. Relatedly, as mentioned above, the identification of intentions presents as a challenge as each infant and their context is unique. While it was possible for observers to reliably agree on an intention within our coding scheme, they relied on a conservative approach with predetermined assumptions to categorize these interactions. In instances where unambiguous contextual evidence for a particular objective was lacking, intentions were categorized as expressive, guided by affiliative cues such as gaze, affect, or play. Conversely, the classification of intentions as either requestive, information‐seeking or information giving necessitated more substantial supporting evidence, incorporating factors such as persistence, repetition, and the child’s response to the caregiver. Future research should aim to better understand how adults perceive communicative intentions of young children's behavior.

In conclusion, this study provides a panoramic picture of child‐initiated communicative bids with four key intentions across the second year of a child's life. Toddlers emerge are active communicators, initiating interactions to fulfill various communicative goals, including information seeking and information giving, not previously systematically characterized in naturalistic settings.

## Author Contributions

**Didar Karadağ:** conceptualization, investigation, writing – original draft, methodology, visualization, formal analysis, data curation. **Gert Westermann:** conceptualization, methodology, writing – review and editing, supervision. **Marina Bazhydai:** conceptualization, investigation, methodology, writing – review and editing, supervision.

## Funding

This research was supported by the Doctoral Research Program in the Department of Psychology at Lancaster University.

## Ethics Statement

The present study was conducted according to guidelines laid down in the Declaration of Helsinki, with written informed consent obtained from a parent or guardian for each child before any assessment or data collection by the original data owners. We received ethics approval (FST20112, dated May 11, 2021) from the Faculty of Science and Technology Research Ethics Committee at Lancaster University in the United Kingdom for the behavioral coding and re‐analysis of the primary dataset.

## Conflicts of Interest

The authors declare no conflicts of interest.

## Data Availability

Anonymized coded data can be accessed on Open Science Framework here (https://osf.io/b93r5/?view_only=d49caef0281948a6b7d0c5086ae36c50). The analyses presented here were not preregistered.

## Endnotes

^1^
Given the nature of the data, we were not able to calculate a Kappa value for the event detection.

## References

[infa70072-bib-0001] Ashley, J. , and M. Tomasello . 1998. “Cooperative Problem‐Solving and Teaching in Preschoolers.” Social Development 7, no. 2: 143–163. 10.1111/1467-9507.00059.

[infa70072-bib-0002] Aureli, T. , P. Perucchini , and J. M. Iverson . 2013. “Motor Acts and Communicative Gestures From 9 to 18 Months of Age in Imperative and Declarative Context: Tracing the Origin and Development of Pointing.” Rivista di psicolinguistica applicata: XIII 2: 21–30. https://www.torrossa.com/en/resources/an/2908531?digital=true.

[infa70072-bib-0003] Aureli, T. , M. Spinelli , M. Fasolo , M. C. Garito , P. Perucchini , and L. D’Odorico . 2017. “The Pointing– Vocal Coupling Progression in the First Half of the Second Year of Life.” Infancy 22, no. 6: 801–818. 10.1111/infa.12181.

[infa70072-bib-0004] Bates, E. , L. Camaioni , and V. Volterra . 1975. “The Acquisition of Performatives Prior to Speech.” Merrill‐Palmer Quarterly of Behavior and Development 21, no. 3: 205–226. https://www.jstor.org/stable/23084619.

[infa70072-bib-0005] Bazhydai, M. , and P. L. Harris . 2021. “Infants Actively Seek and Transmit Knowledge via Communication.” Behavioral and Brain Sciences 44: e142. 10.1017/S0140525X20001405. 34796804

[infa70072-bib-0006] Bazhydai, M. , and D. Karadağ . 2022. “Can Bifocal Stance Theory Explain Children’s Selectivity in Active Information Transmission?” Behavioral and Brain Sciences 45: e251. 10.1017/S0140525X22001327. 36353893

[infa70072-bib-0007] Bazhydai, M. , P. Silverstein , E. Parise , and G. Westermann . 2020. “Two‐Year‐Old Children Preferentially Transmit Simple Actions but Not Pedagogically Demonstrated Actions.” Developmental Science 23, no. 5: e12941. 10.1111/desc.12941. 31981382

[infa70072-bib-0008] Bazhydai, M. , G. Westermann , and E. Parise . 2020. “‘I Don’t Know but I Know Who to Ask’: 12‐Month‐Olds Actively Seek Information From Knowledgeable Adults.” Developmental Science 23, no. 5: e12938. 10.1111/desc.12938. 31954092

[infa70072-bib-0009] Begus, K. , T. Gliga , and V. Southgate . 2014. “Infants Learn What They Want to Learn: Responding to Infant Pointing Leads to Superior Learning.” PLoS One 9, no. 10: e108817. 10.1371/journal.pone.0108817. 25290444 PMC4188542

[infa70072-bib-0010] Begus, K. , and V. Southgate . 2012. “Infant Pointing Serves an Interrogative Function.” Developmental Science 15, no. 5: 611–617. 10.1111/j.1467-7687.2012.01160.x. 22925509

[infa70072-bib-0011] Begus, K. , and V. Southgate . 2018. “Curious Learners: How Infants’ Motivation to Learn Shapes and Is Shaped by Infants’ Interactions With the Social World.” In Active Learning From Infancy to Childhood: Social Motivation, Cognition, and Linguistic Mechanisms, 13–37. 10.1007/978-3-319-77182-3_2.

[infa70072-bib-0012] Behne, T. , M. Carpenter , and M. Tomasello . 2014. “Young Children Create Iconic Gestures to Inform Others.” Developmental Psychology 50, no. 8: 2049–2060. 10.1037/a0037224. 24979473

[infa70072-bib-0013] Bensalah, L. , and S. Caillies . 2020. “High and Low Theory‐of‐Mind Scores of Child‐Teachers: Which Teaching Strategies Are Efficient?” Cognitive Development 55: 100920. 10.1016/j.cogdev.2020.100920.

[infa70072-bib-0014] Blake, J. , P. O’Rourke , and G. Borzellino . 1994. “Form and Function in the Development of Pointing and Reaching Gestures.” Infant Behavior and Development 17, no. 2: 195–203. 10.1016/0163-6383(94)90055-8.

[infa70072-bib-0015] Bornstein, M. H. , D. L. Putnick , C. S. Hahn , C. S. Tamis‐LeMonda , and G. Esposito . 2020. “Stabilities of Infant Behaviors and Maternal Responses to Them.” Infancy 25, no. 3: 226–245. 10.1111/infa.12326. 32536831 PMC7291865

[infa70072-bib-0016] Boundy, L. , T. Cameron‐Faulkner , and A. Theakston . 2016. “Exploring Early Communicative Behaviors: A Fine‐Grained Analysis of Infant Shows and Gives.” Infant Behavior and Development 44: 86–97. 10.1016/j.infbeh.2016.06.005. 27336182

[infa70072-bib-0017] Boundy, L. , T. Cameron‐Faulkner , and A. Theakston . 2019. “Intention or Attention Before Pointing: Do Infants’ Early Holdout Gestures Reflect Evidence of a Declarative Motive?” Infancy 24, no. 2: 228–248. 10.1111/infa.12267. 32677199

[infa70072-bib-0018] Burdett, E. R. , L. G. Dean , and S. Ronfard . 2017. “A Diverse and Flexible Teaching Toolkit Facilitates the Human Capacity for Cumulative Culture.” Review of Philosophy and Psychology 9, no. 4: 1–12. 10.1007/s13164-017-0345-4. PMC6290851 30595766

[infa70072-bib-0019] Butler, L. P. 2020. “The Empirical Child? A Framework for Investigating the Development of Scientific Habits of Mind.” Child Development Perspectives 14, no. 1: 34–40. 10.1111/cdep.12354.

[infa70072-bib-0020] Caldwell, C. A. , E. Renner , and M. Atkinson . 2018. “Human Teaching and Cumulative Cultural Evolution.” Review of Philosophy and Psychology 9, no. 4: 751–770. 10.1007/s13164-017-0346-3. 30595765 PMC6290649

[infa70072-bib-0021] Camaioni, L. 1997. “The Emergence of Intentional Communication in Ontogeny, Phylogeny, and Pathology.” European Psychologist 2, no. 3: 216–225. 10.1027/1016-9040.2.3.216.

[infa70072-bib-0022] Camaioni, L. , P. Perucchini , F. Bellagamba , and C. Colonnesi . 2004. “The Role of Declarative Pointing in Developing a Theory of Mind.” Infancy 5, no. 3: 291–308. 10.1207/s15327078in0503_3.

[infa70072-bib-0023] Cameron‐Faulkner, T. 2020. “The Emergence of Gesture During Prelinguistic Interaction.” In Current Perspectives on Child Language Acquisition: How Children Use Their Environment to Learn, Vol. 27, 173–188. John Benjamins Publishing Company.

[infa70072-bib-0024] Cameron‐Faulkner, T. , A. Theakston , E. Lieven , and M. Tomasello . 2015. “The Relationship Between Infant Holdout and Gives and Pointing.” Infancy 20, no. 5: 576–586. 10.1111/infa.12085.

[infa70072-bib-0025] Caro, T. , and M. Hauser . 1992. “Is There Teaching in Nonhuman Animals?” Quarterly Review of Biology 67, no. 2: 151–174. 10.1086/417553. 1635977

[infa70072-bib-0026] Carpendale, J. , and C. Lewis . 2006. How Children Develop Social Understanding. Blackwell Publishing.

[infa70072-bib-0027] Carpendale, J. I. , U. Müller , B. Wallbridge , T. Broesch , T. Cameron‐Faulkner , and K. Ten Eycke . 2021. “The Development of Giving in Forms of Object Exchange: Exploring the Roots of Communication and Morality in Early Interaction Around Objects.” Human Development 65, no. 3: 166–179. 10.1159/000517221.

[infa70072-bib-0028] Carpenter, M. , K. Nagell , M. Tomasello , G. Butterworth , and C. Moore . 1998. “Social Cognition, Joint Attention, and Communicative Competence From 9 to 15 Months of Age.” Monographs of the Society for Research in Child Development 63, no. 4: 1–174. 10.2307/1166214. 9835078

[infa70072-bib-0029] Caselli, M. C. 1990. “Communicative Gestures and First Words.” In From Gesture to Language in Hearing and Deaf Children, 56–67. Springer Berlin Heidelberg.

[infa70072-bib-0030] Choi, B. , R. Wei , and M. L. Rowe . 2021. “Show, Give, and Point Gestures Across Infancy Differentially Predict Language Development.” Developmental Psychology 57, no. 6: 851–862. 10.1037/dev0001195. 34424004 PMC8386023

[infa70072-bib-0031] Chouinard, M. M. , P. L. Harris , and M. P. Maratsos . 2007. “Children’s Questions: A Mechanism for Cognitive Development.” Monographs of the Society for Research in Child Development 72, no. 1: i–129. http://www.jstor.org/stable/30163594. 10.1111/j.1540-5834.2007.00412.x 17394580

[infa70072-bib-0032] Clark, H. H. 1996. Using Language. Cambridge University Press.

[infa70072-bib-0033] Cochet, H. , and J. Vauclair . 2010a. “Pointing Gestures Produced by Toddlers From 15 to 30 Months: Different Functions, Hand Shapes and Laterality Patterns.” Infant Behavior and Development 33, no. 4: 431–441. 10.1016/j.infbeh.2010.04.009. 20546910

[infa70072-bib-0034] Cochet, H. , and J. Vauclair . 2010b. “Features of Spontaneous Pointing Gestures in Toddlers.” Gesture 10, no. 1: 86–107. 10.1075/gest.10.1.05coc.

[infa70072-bib-0035] Dahl, A. , and M. Paulus . 2019. “From Interest to Obligation: The Gradual Development of Human Altruism.” Child Development Perspectives 13, no. 1: 10–14. 10.1111/cdep.12298.

[infa70072-bib-0036] Davis‐Unger, A. C. , and S. M. Carlson . 2008a. “Children’s Teaching Skills: The Role of Theory of Mind and Executive Function.” Mind, Brain, and Education 2, no. 3: 128–135. 10.1111/j.1751-228X.2008.00043.x.

[infa70072-bib-0037] Davis‐Unger, A. C. , and S. M. Carlson . 2008b. “Development of Teaching Skills and Relations to Theory of Mind in Preschoolers.” Journal of Cognition and Development 9, no. 1: 26–45. 10.1080/15248370701836584.

[infa70072-bib-0038] Donnellan, E. , C. Bannard , M. L. McGillion , K. E. Slocombe , and D. Matthews . 2020. “Infants’ Intentionally Communicative Vocalizations Elicit Responses From Caregivers and Are the Best Predictors of the Transition to Language: A Longitudinal Investigation of Infants’ Vocalizations, Gestures and Word Production.” Developmental Science 23, no. 1: e12843. 10.1111/desc.12843. 31045301

[infa70072-bib-0039] ELAN 2023. “ELAN (Version 6.7).” [Computer Software]. Nijmegen: Max Planck Institute for Psycholinguistics, The Language Archive. https://archive.mpi.nl/tla/elan.

[infa70072-bib-0040] Flynn, E. , and A. Whiten . 2012. “Experimental ‘Microcultures’ in Young Children: Identifying Biographic, Cognitive, and Social Predictors of Information Transmission.” Child Development 83, no. 3: 911–925. 10.1111/j.1467-8624.2012.01747.x. 22417384

[infa70072-bib-0041] Goldstein, M. H. , J. Schwade , J. Briesch , and S. Syal . 2010. “Learning While Babbling: Prelinguistic Object‐Directed Vocalizations Indicate a Readiness to Learn.” Infancy 15, no. 4: 362–391. 10.1111/j.1532-7078.2009.00020.x. 32693523

[infa70072-bib-0042] Goupil, L. , M. Romand‐Monnier , and S. Kouider . 2016. “Infants Ask for Help When They Know They Don’t Know.” Proceedings of the National Academy of Sciences 113, no. 13: 3492–3496. 10.1073/pnas.1515129113. PMC4822620 26951655

[infa70072-bib-0043] Greenfield, P. M. , and E. S. Savage‐Rumbaugh . 1993. “Comparing Communicative Competence in Child and Chimp: The Pragmatics of Repetition.” Journal of Child Language 20, no. 1: 1–26. 10.1017/S0305000900009090. 7681067

[infa70072-bib-0044] Grice, H. P. 1975. “Logic and Conversation.” In Speech Acts, 41–58. Brill.

[infa70072-bib-0045] Guevara, I. , and C. Rodríguez . 2023. “Developing Communication Through Objects: Ostensive Gestures as the First Gestures in Children’s Development.” Developmental Review 68: 101076. 10.1016/j.dr.2023.101076.

[infa70072-bib-0046] Guevara, I. , C. Rodríguez , and M. Núñez . 2024. “Developing Gestures in the Infant Classroom: From Showing and Giving to Pointing.” European Journal of Psychology of Education 39, no. 4: 4671–4702. 10.1007/s10212-024-00895-6.

[infa70072-bib-0047] Harris, P. L. , L. Butler , S. Ronfard , and K. Corriveau . 2020. “The Point, the Shrug, and the Question of Clarification.” In The Questioning Child: Insights from Psychology and Education, edited by L. Butler , S. Ronfard , and K. Corriveau , 29–50.

[infa70072-bib-0048] Harris, P. L. , and J. D. Lane . 2014. “Infants Understand How Testimony Works.” Topoi 33, no. 2: 443–458. 10.1007/s11245-013-9180-0. 35874967 PMC9306287

[infa70072-bib-0049] Heinsberg, L. W. , and D. E. Weeks . 2022. “Post Hoc Power Is Not Informative.” Genetic Epidemiology 46, no. 7: 390–394. 10.1002/gepi.22464. 35642557 PMC9452450

[infa70072-bib-0050] Herzberg, O. , K. K. Fletcher , J. L. Schatz , K. E. Adolph , and C. S. Tamis‐LeMonda . 2022. “Infant Exuberant Object Play at Home: Immense Amounts of Time‐Distributed, Variable Practice.” Child Development 93, no. 1: 150–164. 10.1111/cdev.13669. 34515994 PMC8974536

[infa70072-bib-0051] Karadağ, D. , M. Bazhydai , S. Koşkulu‐Sancar , and H. H. Şen . 2024. “The Breadth and Specificity of 18‐Month‐Old’s Infant‐Initiated Interactions in Naturalistic Home Settings.” Infant Behavior and Development 74: 101927. 10.1016/j.infbeh.2024.101927. 38428279

[infa70072-bib-0052] Karadağ, D. , M. Bazhydai , and G. Westermann . 2024. “Toddlers Do Not Preferentially Transmit Generalisable Information to Others.” Developmental Science 27, no. 4: e13479. 10.1111/desc.13479. 38327112

[infa70072-bib-0053] Karadağ, D. , M. Bazhydai , and G. Westermann . 2025. “Young Children’s Transmission of Information Following Self‐Discovery and Instruction.” Frontiers in Developmental Psychology 3: 1553491. 10.3389/fdpys.2025.1553491.

[infa70072-bib-0054] Karasik, L. B. , C. S. Tamis‐LeMonda , and K. E. Adolph . 2011. “Transition From Crawling to Walking and Infants’ Actions With Objects and People.” Child Development 82, no. 4: 1199–1209. 10.1111/j.1467-8624.2011.01595.x. 21545581 PMC3163171

[infa70072-bib-0055] Kishimoto, T. , Y. Shizawa , J. Yasuda , T. Hinobayashi , and T. Minami . 2007. “Do Pointing Gestures by Infants Provoke Comments From Adults?” Infant Behavior and Development 30, no. 4: 562–567. 10.1016/j.infbeh.2007.04.001. 17561263

[infa70072-bib-0056] Knudsen, B. , and U. Liszkowski . 2012a. “Eighteen‐and 24‐Month‐Old Infants Correct Others in Anticipation of Action Mistakes.” Developmental Science 15, no. 1: 113–122. 10.1111/j.1467-7687.2011.01098.x. 22251297

[infa70072-bib-0057] Knudsen, B. , and U. Liszkowski . 2012b. “18‐Month‐Olds Predict Specific Action Mistakes Through Attribution of False Belief, Not Ignorance, and Intervene Accordingly.” Infancy 17, no. 6: 672–691. 10.1111/j.1532-7078.2011.00105.x. 32693489

[infa70072-bib-0058] Kuchirko, Y. , L. Tafuro , and C. S. Tamis LeMonda . 2018. “Becoming a Communicative Partner: Infant Contingent Responsiveness to Maternal Language and Gestures.” Infancy 23, no. 4: 558–576. 10.1111/infa.12222.

[infa70072-bib-0059] Kuhn, L. J. , M. T. Willoughby , M. P. Wilbourn , L. Vernon‐Feagans , C. B. Blair , and Family Life Project Key Investigators . 2014. “Early Communicative Gestures Prospectively Predict Language Development and Executive Function in Early Childhood.” Child Development 85, no. 5: 1898–1914. https://srcd.onlinelibrary.wiley.com/doi/10.1111/cdev.12249. 24773289 10.1111/cdev.12249 PMC4165687

[infa70072-bib-0060] Kulke, L. , M. Reiß , H. Krist , and H. Rakoczy . 2018. “How Robust Are Anticipatory Looking Measures of Theory of Mind? Replication Attempts Across the Life Span.” Cognitive Development 46: 97–111. 10.1016/j.cogdev.2017.09.001.

[infa70072-bib-0061] Lakens, D. 2022. “Sample Size Justification.” Collabra: Psychology 8, no. 1: 33267. 10.1525/collabra.33267.

[infa70072-bib-0062] LeBarton, E. S. , S. Goldin‐Meadow , and S. Raudenbush . 2015. “Experimentally Induced Increases in Early Gesture Lead to Increases in Spoken Vocabulary.” Journal of Cognition and Development 16, no. 2: 199–220. 10.1080/15248372.2013.858041. 26120283 PMC4480788

[infa70072-bib-0063] Liszkowski, U. , M. Carpenter , A. Henning , T. Striano , and M. Tomasello . 2004. “Twelve‐Month‐Olds Point to Share Attention and Interest.” Developmental Science 7, no. 3: 297–307. 10.1111/j.1467-7687.2004.00349.x. 15595371

[infa70072-bib-0064] Liszkowski, U. , M. Carpenter , T. Striano , and M. Tomasello . 2006. “12‐and 18‐Month‐Olds Point to Provide Information for Others.” Journal of Cognition and Development 7, no. 2: 173–187. 10.1207/s15327647jcd0702_2.

[infa70072-bib-0065] Liszkowski, U. , M. Carpenter , and M. Tomasello . 2008. “Twelve‐Month‐Olds Communicate Helpfully and Appropriately for Knowledgeable and Ignorant Partners.” Cognition 108, no. 3: 732–739. 10.1016/j.cognition.2008.06.013. 18721918

[infa70072-bib-0066] Lucca, K. 2020. “The Development of Information‐Requesting Gestures in Infancy and Their Role in Shaping Learning Outcomes.” In The Questioning Child: Insights from Psychology and Education, edited by L. P. Butler , S. Ronfard , and K. H. Corriveau , 89–117. Cambridge University Press. 10.1017/9781108553803.006.

[infa70072-bib-0067] Lucca, K. , and M. P. Wilbourn . 2018. “Communicating to Learn: Infants’ Pointing Gestures Result in Optimal Learning.” Child Development 89, no. 3: 941–960. 10.1111/cdev.12707. 28032638

[infa70072-bib-0068] Lucca, K. , and M. P. Wilbourn . 2019. “The What and the How: Information‐Seeking Pointing Gestures Facilitate Learning Labels and Functions.” Journal of Experimental Child Psychology 178: 417–436. 10.1016/j.jecp.2018.08.003. 30318380

[infa70072-bib-0069] Meng, X. , and K. Hashiya . 2014. “Pointing Behavior in Infants Reflects the Communication Partner’s Attentional and Knowledge States: A Possible Case of Spontaneous Informing.” PLoS One 9, no. 9: 1–8. 10.1371/journal.pone.0107579. PMC4161458 25211279

[infa70072-bib-0070] Michael, J. , A. Green , B. Siposova , K. Jensen , and S. Kita . 2022. “Finish What You Started: 2‐Year‐Olds Motivated by a Preference for Completing Others’ Unfinished Actions in Instrumental Helping Contexts.” Cognitive Science 46, no. 6: e13160. 10.1111/cogs.13160. 35665955

[infa70072-bib-0071] Moore, C. , and B. D’Entremont . 2001. “Developmental Changes in Pointing as a Function of Attentional Focus.” Journal of Cognition and Development 2, no. 2: 109–129. 10.1207/S15327647JCD0202_1.

[infa70072-bib-0072] Moreno‐Núñez, A. , C. Rodríguez , and E. Miranda‐Zapata . 2020. “Getting away From the Point: The Emergence of Ostensive Gestures and Their Functions.” Journal of Child Language 47, no. 3: 556–578. 10.1017/S0305000919000606. 31685056

[infa70072-bib-0073] Oakes, L. M. 2009. “The ‘Humpty Dumpty Problem’ in the Study of Early Cognitive Development: Putting the Infant Back Together Again.” Perspectives on Psychological Science 4, no. 4: 352–358. 10.1111/j.1745-6924.2009.01137.x. 20161394 PMC2782855

[infa70072-bib-0074] Oakes, L. M. 2023. “Understanding Developmental Cascades and Experience: Diversity Matters.” Infancy 28, no. 3: 492–506. 10.1111/infa.12539. 36961430

[infa70072-bib-0075] Olson, J. , and E. F. Masur . 2013. “Mothers Respond Differently to Infants’ Gestural Versus Nongestural Communicative Bids.” First Language 33, no. 4: 372–387. 10.1177/0142723713493346.

[infa70072-bib-0076] O’Neill, D. K. 1996. “Two‐Year‐Old Children’s Sensitivity to a Parent’s Knowledge State when Making Requests.” Child Development 67, no. 2: 659–677. 10.1111/j.1467-8624.1996.tb01758.x.

[infa70072-bib-0077] Paulus, M. 2020. “Is Young Children’s Helping Affected by Helpees’ Need? Preschoolers, but Not Infants Selectively Help Needy Others.” Psychological Research 84, no. 5: 1440–1450. 10.1007/s00426-019-01148-8. 30758652 PMC7270991

[infa70072-bib-0078] Perucchini, P. , A. Bello , F. Presaghi , and T. Aureli . 2021. “Developmental Trajectories in Infant Pointing: The Effects of Vocalisation and Communicative Intention.” First Language 41, no. 3: 314–335. 10.1177/0142723720980196.

[infa70072-bib-0079] Poulin‐Dubois, D. , H. Rakoczy , K. Burnside , et al. 2018. “Do Infants Understand False Beliefs? We Don’t Know Yet–A Commentary on Baillargeon, Buttelmann and Southgate’s Commentary.” Cognitive Development 48: 302–315. 10.1016/j.cogdev.2018.09.005.

[infa70072-bib-0080] Premack, S. , and A. J. Premack . 1983. The Mind of an Ape. Norton.

[infa70072-bib-0081] Qiu, F. W. , J. Park , A. Vite , E. Patall , and H. Moll . 2024. “Children’s Selective Teaching and Informing: A Meta‐Analysis.” Developmental Science 28, no. 1: e13576. 10.1111/desc.13576. 39380203

[infa70072-bib-0082] Ramenzoni, V. C. , and U. Liszkowski . 2016. “The Social Reach: 8‐Month‐Olds Reach for Unobtainable Objects in the Presence of Another Person.” Psychological Science 27, no. 9: 1278–1285. 10.1177/0956797616659938. 27481910

[infa70072-bib-0083] Rivas, E. 2005. “Recent Use of Signs by Chimpanzees (Pan Troglodytes) in Interaction With Humans.” Journal of Comparative Psychology 119, no. 4: 404–417. 10.1037/0735-7036.119.4.404. 16366774

[infa70072-bib-0084] Rochat, P. , N. Goubet , and S. J. Senders . 1999. “To Reach or Not to Reach? Perception of Body Effectivities by Young Infants.” Infant and Child Development: An International Journal of Research and Practice 8, no. 3: 129–148. 10.1002/(sici)1522-7219(199909)8:3<129::aid-icd193>3.0.co;2-g.

[infa70072-bib-0085] Rohlfing, K. J. , C. Lüke , U. Liszkowski , U. Ritterfeld , and A. Grimminger . 2022. “Developmental Paths of Pointing for Various Motives in Infants With and Without Language Delay.” International Journal of Environmental Research and Public Health 19, no. 9: 4982. 10.3390/ijerph19094982. 35564377 PMC9104230

[infa70072-bib-0086] Ronfard, S. , I. M. Zambrana , T. K. Hermansen , and D. Kelemen . 2018. “Question‐Asking in Childhood: A Review of the Literature and a Framework for Understanding Its Development.” Developmental Review 49: 101–120. 10.1016/j.dr.2018.05.002.

[infa70072-bib-0087] Rowe, M. L. , and K. A. Leech . 2019. “A Parent Intervention With a Growth Mindset Approach Improves Children’s Early Gesture and Vocabulary Development.” Developmental Science 22, no. 4: e12792. 10.1111/desc.12792. 30570813 PMC7041843

[infa70072-bib-0088] Ruether, J. , and U. Liszkowski . 2024. “Ontogeny of Index‐Finger Pointing.” Journal of Child Language 51, no. 5: 1050–1066. 10.1017/S0305000923000053. 36722255

[infa70072-bib-0089] Salo, V. C. , B. Reeb‐Sutherland , T. I. Frenkel , L. C. Bowman , and M. L. Rowe . 2019. “Does Intention Matter? Relations Between Parent Pointing, Infant Pointing, and Developing Language Ability.” Journal of Cognition and Development 20, no. 5: 635–655. 10.1080/15248372.2019.1648266. 32089652 PMC7034940

[infa70072-bib-0090] Salter, G. , and M. Carpenter . 2022. “Showing and Giving: From Incipient to Conventional Forms.” Philosophical Transactions of the Royal Society B 377, no. 1859: 20210102. 10.1098/rstb.2021.0102. PMC9310177 35876202

[infa70072-bib-0091] Schneider, J. L. , and J. M. Iverson . 2022. “Cascades in Action: How the Transition to Walking Shapes Caregiver Communication During Everyday Interactions.” Developmental Psychology 58, no. 1: 1–16. 10.1037/dev0001280. 34843275 PMC9588170

[infa70072-bib-0092] Slone, L. K. , L. B. Smith , and C. Yu . 2019. “Self‐Generated Variability in Object Images Predicts Vocabulary Growth.” Developmental Science 22, no. 6: e12816. 10.1111/desc.12816. 30770597 PMC6697249

[infa70072-bib-0093] Southgate, V. , C. Van Maanen , and G. Csibra . 2007. “Infant Pointing: Communication to Cooperate or Communication to Learn?” Child Development 78, no. 3: 735–740. 10.1111/j.1467-8624.2007.01028.x. 17517000

[infa70072-bib-0094] Strauss, S. , C. I. Calero , and M. Sigman . 2014. “Teaching, Naturally.” Trends in Neuroscience and Education 3, no. 2: 38–43. 10.1016/j.tine.2014.05.001.

[infa70072-bib-0095] Strauss, S. , and M. Ziv . 2012. “Teaching Is a Natural Cognitive Ability for Humans.” Mind, Brain, and Education 6, no. 4: 186–196. 10.1111/j.1751-228X.2012.01156.x.

[infa70072-bib-0096] Strauss, S. , M. Ziv , and A. Stein . 2002. “Teaching as a Natural Cognition and Its Relations to Preschoolers’ Developing Theory of Mind.” Cognitive Development 17, no. 3–4: 1473–1487. 10.1016/S0885-2014(02)00128-4.

[infa70072-bib-0097] Striano, T. , A. Vaish , and J. P. Benigno . 2006. “The Meaning of Infants’ Looks: Information Seeking and Comfort Seeking?” British Journal of Developmental Psychology 24, no. 3: 615–630. 10.1348/026151005X67566.

[infa70072-bib-0098] Suarez‐Rivera, C. , K. K. Fletcher , and C. S. Tamis‐LeMonda . 2024. “Infants’ Home Auditory Environment: Background Sounds Shape Language Interactions.” Developmental Psychology 60, no. 12: 2274–2289. 10.1037/dev0001762. 39325385 PMC11965805

[infa70072-bib-0099] Suarez‐Rivera, C. , J. L. Schatz , O. Herzberg , and C. S. Tamis‐LeMonda . 2022. “Joint Engagement in the Home Environment Is Frequent, Multimodal, Timely, and Structured.” Infancy 27, no. 2: 232–254. 10.1111/infa.12446. 34990043

[infa70072-bib-0100] Surian, L. , S. Caldi , and D. Sperber . 2007. “Attribution of Beliefs by 13‐Month‐Old Infants.” Psychological Science 18, no. 7: 580–586. 10.1111/j.1467-9280.2007.01943.x. 17614865

[infa70072-bib-0101] Swirbul, M. S. , O. Herzberg , and C. S. Tamis‐LeMonda . 2022. “Object Play in the Everyday Home Environment Generates Rich Opportunities for Infant Learning.” Infant Behavior and Development 67: 101712. 10.1016/j.infbeh.2022.101712. 35378342

[infa70072-bib-0102] Tamis‐LeMonda, C. , and K. Adolph . 2017. “The Science of Everyday Play.” Databrary. https://nyu.databrary.org/volume/563.

[infa70072-bib-0103] Tamis‐LeMonda, C. S. 2023. “The Mountain Stream of Infant Development.” Infancy 28, no. 3: 468–491. 10.1111/infa.12538. 36961322 PMC10184132

[infa70072-bib-0104] Tamis‐LeMonda, C. S. , M. H. Bornstein , R. Kahana‐Kalman , L. Baumwell , and L. Cyphers . 1998. “Predicting Variation in the Timing of Language Milestones in the Second Year: An Events History Approach.” Journal of Child Language 25, no. 3: 675–700. 10.1017/S0305000998003572. 10095330

[infa70072-bib-0105] Tamis‐Lemonda, C. S. , Y. Kuchirko , and L. Tafuro . 2013. “From Action to Interaction: Infant Object Exploration and Mothers’ Contingent Responsiveness.” IEEE Transactions on Autonomous Mental Development 5, no. 3: 202–209. 10.1109/TAMD.2013.2269905beyb.

[infa70072-bib-0106] Tomasello, M. , M. Carpenter , and U. Liszkowski . 2007. “A New Look at Infant Pointing.” Child Development 78, no. 3: 705–722. 10.1111/j.1467-8624.2007.01025.x. 17516997

[infa70072-bib-0107] Tomasello, M. , and K. Haberl . 2003. “Understanding Attention: 12‐and 18‐Month‐Olds Know What Is New for Other Persons.” Developmental Psychology 39, no. 5: 906–912. 10.1037/0012-1649.39.5.906. 12952402

[infa70072-bib-0108] Vredenburgh, C. , T. Kushnir , and M. Casasola . 2015. “Pedagogical Cues Encourage Toddlers’ Transmission of Recently Demonstrated Functions to Unfamiliar Adults.” Developmental Science 18, no. 4: 645–654. 10.1111/desc.12233. 25284008

[infa70072-bib-0109] Wittenburg, P. , H. Brugman , A. Russel , A. Klassmann , and H. Sloetjes . 2006. “ELAN: A Professional Framework for Multimodality Research.” In 5th International Conference on Language Resources and Evaluation (LREC 2006), 1556–1559.

[infa70072-bib-0110] Yu, Y. , E. Bonawitz , and P. Shafto . 2019. “Pedagogical Questions in Parent–Child Conversations.” Child Development 90, no. 1: 147–161. 10.1111/cdev.12850. 28617972

[infa70072-bib-0111] Zhang, Y. , R. Hedo , A. Rivera , R. Rull , S. Richardson , and X. M. Tu . 2019. “Post Hoc Power Analysis: Is It an Informative and Meaningful Analysis?” General Psychiatry 32, no. 4: e100069. 10.1136/gpsych-2019-100069. 31552383 PMC6738696

[infa70072-bib-0112] Ziv, M. , A. Solomon , S. Strauss , and D. Frye . 2016. “Relations Between the Development of Teaching and Theory of Mind in Early Childhood.” Journal of Cognition and Development 17, no. 2: 264–284. 10.1080/15248372.2015.1048862.

